# Monitoring the effect of first line treatment in RAS/RAF mutated metastatic colorectal cancer by serial analysis of tumor specific DNA in plasma

**DOI:** 10.1186/s13046-018-0723-5

**Published:** 2018-03-12

**Authors:** C. B. Thomsen, T. F. Hansen, R. F. Andersen, J. Lindebjerg, L. H. Jensen, A. Jakobsen

**Affiliations:** 1 Danish Colorectal Cancer Center South, Vejle Hospital, Beriderbakken 4, DK-7100 Vejle, Denmark; 2 Institute of Regional Health Research, University of Southern Denmark, Odense, Denmark

**Keywords:** Circulating tumor DNA, RAS/RAF mutations, Metastatic colorectal cancer, Liquid biopsy, Monitoring, Treatment efficiency

## Abstract

**Background:**

Precision medicine calls for an early indicator of treatment efficiency. Circulating tumor DNA (ctDNA) is a promising marker in this setting. Our prospective study explored the association between disease development and change of ctDNA during first line chemotherapy in patients with RAS/RAF mutated metastatic colorectal cancer (mCRC).

**Methods:**

The study included 138 patients with mCRC receiving standard first line treatment. In patients with RAS/RAF mutated tumor DNA the same mutation was quantified in the plasma using droplet digital PCR. The fractional abundance of ctDNA was assessed in plasma before treatment start and at every treatment cycle until radiologically defined progressive disease.

**Results:**

RAS/RAF mutations were detected in the plasma from 77 patients. Twenty patients progressed on treatment and 57 stopped treatment without progression. The presence of mutated DNA in plasma was correlated with poor overall survival. A low level of ctDNA after the first cycle of chemotherapy was associated with a low risk of progression. On the other hand, a significant increase of ctDNA at any time during the treatment course was associated with a high risk of progression on continuous treatment. The first increase in ctDNA level occurred at a median of 51 days before radiologically confirmed progression.

**Conclusions:**

The results indicate that the ctDNA level holds potential as a clinically valuable marker in first line treatment of mCRC. A rapid decrease was associated with a prolonged progression free interval, whereas a significant increase gave notice of early progression with a relevant lead time.

**Electronic supplementary material:**

The online version of this article (10.1186/s13046-018-0723-5) contains supplementary material, which is available to authorized users.

## Background

Precision medicine is an important subject with growing clinical interest in cancer treatment. The concept may be complicated, but its core is as simple as “the right treatment to the right patient at the right time”. The issue has two aspects. Giving the right treatment is essential, but discontinuing ineffective and potentially toxic treatment holds the same importance and places monitoring in a central position.

Easily accessible biomarkers validated for clinical application are warranted. Blood samples have several advantages over tissue biopsies and consequently, the “liquid biopsy” concept has seen increased interest [1].

Tumor specific mutations in tissue rank high as tumor markers of clinical relevance and are detectable by Next Generation Sequencing (NGS). However a complete characterization takes considerable resources and the sensitivity is low, around 2% [2].

In blood a promising approach is analysis of circulating tumor DNA (ctDNA) by droplet digital PCR (ddPCR) with a high sensitivity (> 0.01%).

RAS/RAF mutations occur in 50–60% of colorectal cancer (CRC) patients [3, 4] and predict resistance to EGFR-inhibiting antibodies. In the clinic the mutation analysis thus serves as a tool for selection of targeted treatment. New data show that ctDNA may replace tumor tissue analysis [5–8], and serial analysis may provide information as to the relation of ctDNA with treatment effect and progression [9]. There are few studies in solid tumors dealing with this issue. A proof of concept study has indicated that fluctuations in the ctDNA level reflect changes of tumor size in metastatic breast cancer [10]. A recent study has looked at mutational dynamics during anti-EGFR therapy [11], but there are no studies dealing with prediction of progression in CRC during a treatment course.

The aim of the present study was to investigate the possible value of ctDNA monitoring during chemotherapy with respect to efficiency of first line treatment of metastatic CRC (mCRC).

## Methods

### Study population

Patients with mCRC receiving first line treatment were offered inclusion in a prospective biomarker study at Danish Colorectal Cancer Center South, Vejle Hospital, Denmark. The inclusion criteria are listed in the supplementary material. Plasma samples from all patients were collected at baseline and before every treatment cycle throughout the treatment course. Treatment effect was evaluated at every third cycle according to the RECIST criteria blinded of mutational status.

The study was approved by The Regional Committees on Health Research Ethics for Southern Denmark (S-20100005) and the investigation was conducted in accordance with the REMARK criteria [12].

### Tumor analysis

DdPCR (Bio-Rad®, Hercules, CA, USA) was used for the analysis of all mutations in both tissue and plasma as described below. The mutations were selected by combining the results from two previous studies [13, 14]. The analyses were performed in three consecutive rounds (Additional file 1: Table S1) as previously described [15] and are further described in Additional file 2: Supplementary material.

### Blood sample analysis

Patients with RAS/RAF mutated DNA in the tumor were selected for plasma analysis. We investigated the 26 most frequent KRAS and NRAS mutations in CRC and the most frequent RAF mutation, BRAF V600E, as further described in the supplementary material (Additional file 3: Table S2) [13, 14]. The blood sampled at each cycle of treatment was analyzed for the specific mutation detected in the tumor tissue. A detailed description of the blood sample analysis and the ddPCR can be found in Additional file 2: Supplementary material.

We have recently published a method allowing for screening with subsequent quantitative analysis of 27 RAS/RAF mutations in the blood [16]. This method was applied in the screening of RAS/RAF mutations at time of progression in patients with a wild-type tumor.

The specific mutations and the distribution found in the plasma at baseline are listed in Additional file 4: Table S3.

### Data management

The ctDNA level was defined as the fraction of ctDNA and expressed as the proportion of mutant alleles in the total circulating cell-free DNA (mutated DNA/(mutated DNA + wild-type DNA) and calculated by the Quantasoft software. The variation of the ctDNA level is reported as a 95% confidence interval (95% CI) based on Poisson statistics.

A substantial increase of the ctDNA level (HctDNA) during the treatment course was defined as a value with no overlap between the 95% CI of the current and immediately preceding measurements.

Low ctDNA (LctDNA) after the first treatment cycle was defined as a level with the lower 95% CI overlapping zero.

### Statistical analysis

Full description is given in the Additional file 2: Supplementary material.

## Results

**Fig. 1:**
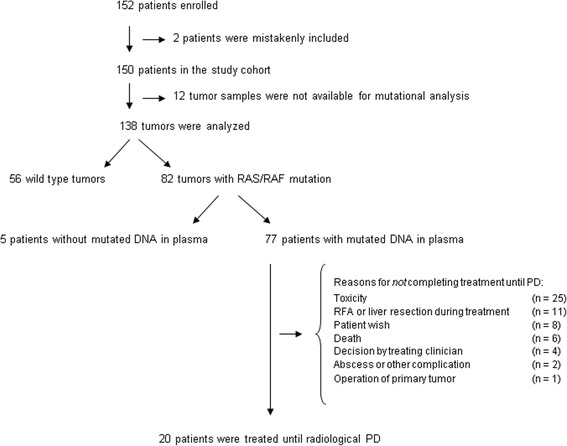
Flow chart

Between March 2010 and November 2015, 152 patients were enrolled in the study. The patient flow is shown in Fig. 1. One patient withdrew his consent and one suffered from another malignant disease and was mistakenly enrolled leaving 150 patients for investigation. Analysis of mutational status was not possible in 12 patients (8%) due to limited access to tumor tissue or insufficient material in the specimens. Thus data were available from 138 patients (92%). Eighty-two patients (59%) had a tumor RAS/RAF mutation and in 77 patients (94%) the mutation was found in the plasma. Baseline patient characteristics of patients with mutated DNA in plasma are shown in Table 1. There was no statistically significant difference in terms of age, gender, and tumor location between the group of patients with wild-type DNA in the plasma and patients with mutated DNA (data not shown). The 77 patients had at least a baseline blood sample drawn and contributed with a total of 571 analyzed blood samples. Twenty patients progressed on treatment (26%), one of which only had a baseline blood sample. Fifty-seven patients stopped treatment without progressive disease (PD) (74%).

**Table 1:** Baseline characteristics of patients with mutated DNA in plasma (*N =* 77)

	All patients with mutated DNA in plasma
Parameter	N (%)
Age, years	
Median (range)	66 (35–79)
Gender	
Female	29 (38)
Male	48 (62)
PS	
0–1	73 (95)
2	4 (5)
Location of primary tumor	
Right/transverse colon	25 (32)
Left colon	21 (27)
Rectum	31 (40)
Primary disseminated disease	63 (81)
Number of metastatic sites	
≤ 2	53 (69)
> 2	24 (31)
Mutations in plasma DNA	
KRAS	64 (83)
NRAS	3 (4)
BRAF	10 (13)

Abbreviations: *N*, number; *PS*, performance status. Some percentages do not add up to 100% due to rounding

The time-of-progression plasma sample from patients with a wild-type tumor was screened for RAS/RAF mutations. All patients were plasma wild-type except one (1/21), whose blood samples were not included in the analysis.

### Effect of plasma mutational status

**Fig. 2:**
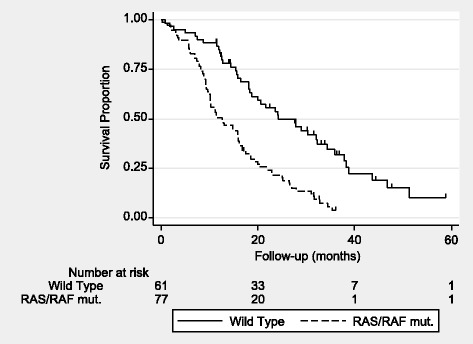
Overall survival according to mutational status The difference is statistically significant, HR = 2.50, 95%CI = 1.69–3.71, *P* < 0.001.Tick marks at censor points.

The median follow-up time was 35.3 months as estimated by inverse Kaplan-Meier analysis. The overall survival (OS) analysis was based on 114 events in the whole cohort of 138 patients of which 124 had at least one response evaluation. Based on tissue samples the best overall response rate (partial response or complete response) was 29%, 18% and 59% in RAS mutated patients, BRAF mutated patients, and wild type patients, respectively. In plasma the same rates were 33%, 20% and 52%, respectively. The difference in response rate between patients with mutated DNA and wild type in plasma translated into a significant difference in progression free survival (PFS). The median PFS was 5.7 months (range 4.2–7.0 months) and 7.8 months (range 6.9–9.9 months), respectively, hazard ratio (HR) = 2.02, 95%CI = 1.38–2.95, *P* < 0.001. A significant difference also applied to OS. The median values were 12.7 months (range 10.2–16.0 months) and 24.2 months (range 18.4–34.3 months), respectively, HR = 2.50, 95%CI = 1.69–3.71, *P* < 0.001 (Fig. 2). Median PFS was 6.2 months (range 4.4–7.2 months) and median OS was 14.8 months (range 10.2–16.8 months) for plasmatic RAS mutated patients alone (*n* = 67).

### Effect of ctDNA level at baseline

Among the 77 patients with tumor and plasma mutation, the ctDNA level at baseline ranged between 0.0026 and 80.43% of the total cell free DNA. The results indicated a clear correlation between the ctDNA level as divided according to the median value and PFS (HR = 3.95, *P =* 0.017). Further calculation on the quantitative importance of the ctDNA level was analyzed by logistic regression. Increased ctDNA at baseline was clearly associated with an increased risk of progression on treatment (relative risk (RR) = 1.02, 95%CI = 1.01–1.03, *P* < 0.001).

### Treatment related dynamics of ctDNA level

**Fig. 3:**
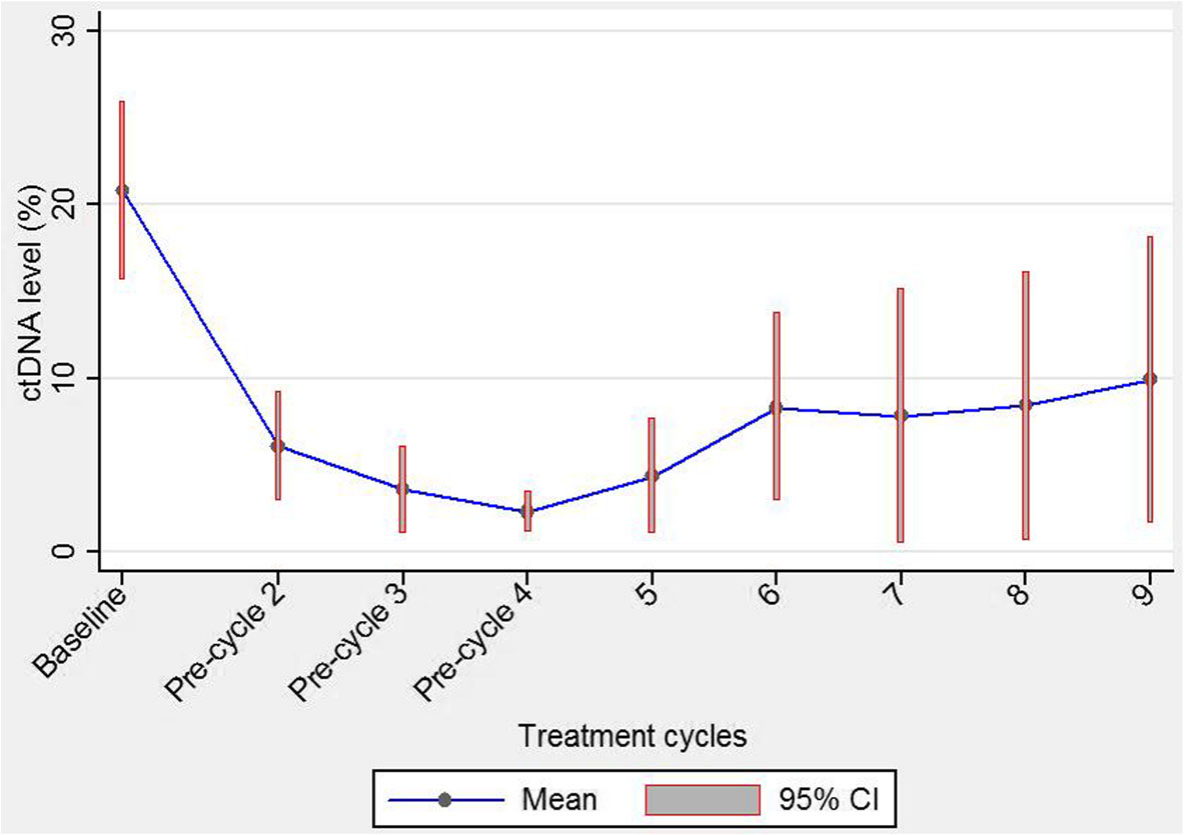
Observed variation in fractional abundance (ctDNA level) ctDNA level is shown over the whole treatment course in all 77 patients with plasma ctDNA. The figure gives mean value and 95%CI.

Figure 3 shows fluctuation of ctDNA level during the treatment course up to nine cycles. It only includes patients on treatment censored at discontinuation irrespective of reason. It is obvious that the ctDNA level decreases rapidly during the first three cycles of treatment from around 20% at baseline to nadir values of approximately 3% before cycle four. The confidence intervals during these and the few subsequent cycles were rather narrow, but they widened towards the third evaluation (pre-cycle nine). The reason was mainly a reduced number of patients for observation but also an increasing fraction of progressing patients with a broad range of ctDNA values.

**Fig. 4:**
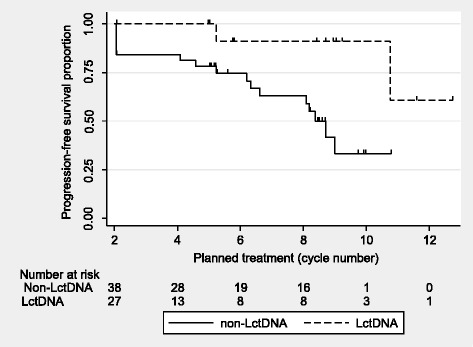
Progression free survival PFS during treatment according to ctDNA at end of cycle one until end of treatment. The difference is statistically significant, HR = 0.16, *P =* 0.017. Tick marks at censor points.

The figure also shows an abrupt decrease of ctDNA after start of treatment. LctDNA was found in 40% (27/65) after the first cycle of chemotherapy (before cycle 2). There was a strong correlation between LctDNA and risk of progression during treatment. Only two patients out of 27 (7%) progressed in the LctDNA group compared to 17/38 (45%) in the other group (non-LctDNA) (*p =* 0.001). This relationship is further underlined in the analysis of PFS during treatment (Fig. 4). The difference according to ctDNA status was statistically significant (HR = 0.16, *P =* 0.017).

HctDNA at any point during the treatment course was highly related to progression on treatment (RR = 4.58, 95%CI = 1.99–10.51, *P* < 0.001). A consecutive HctDNA resulted in a similar high risk of progression (RR = 4.03, 95%CI = 2.11–7.70, *P* < 0.001). The increased level appearing before progression was found by CT scan with a median lead time of 51 days (range 14–133).

## Discussion

In this prospective study of mCRC patients receiving first line chemotherapy we observed a strong correlation between the ctDNA level and clinical endpoints.

Chemotherapy is the corner stone in mCRC treatment. Modern combinations of two or three drugs have improved survival but the treatment still has severe limitations. Firstly, it is only beneficial to two thirds of the patients and secondly, it has serious toxicity. The current methods for monitoring mCRC treatment are far from ideal. The only recommended evaluation modality is imaging [17]. However, CT scanning has drawbacks as to tumor size and volume and is not applicable for small metastases (< 10 mm). Furthermore, progression according to the RECIST criteria calls for at least a 20% size increase.

Therefore, the identification of markers able to guide the treatment holds high priority, especially biomarkers allowing for discontinuation of ineffective treatment at an early time. An indicator in the context of treatment effect should be easily accessible for repeated measurement, which in practice excludes tissue markers. Also, it requires high specificity and sensitivity. Somatic tumor mutations seem to be adequate for this purpose with ctDNA being highly specific. The literature, however, is still sparse.

An approach based on ddPCR has high sensitivity and seems to be in accordance with other techniques described in the literature [18, 19]. It is also highly reproducible as shown in our recent paper [20]. We here present results of serial measurements over a whole treatment course in patients with RAS/RAF mutated tumors. Mutated DNA was detected in the blood stream in > 90% of the patients. This is in agreement with several other studies [21, 22].

We chose to investigate the clinical value of the fractional abundance of ctDNA, since previous studies have focused on this quantity as a central measure in the prognostic and predictive setting [21, 23]. The interpretation of ctDNA data in a clinical context calls for careful consideration and at present there is no general agreement as to quantitative classification related to clinical parameters. Relative reduction of ctDNA level has limitations, since it does not account for low baseline levels. An absolute value in ng/ml or copies/ml seems more attractive. We chose the 95% CI, which is a simple and unique measure. It is easily calculated and seems relevant to account for different values at different time points over a long treatment course.

The prognostic value of ctDNA in mCRC patients has previously been evaluated. Recent studies found a marked prognostic value of ctDNA in OS estimates [24, 25]. Our result supports their conclusion, as patients with a plasma mutation were shown to have a significantly worse survival compared to the rest of the cohort (Fig. 2). We also found that patients presenting with mutated DNA in plasma had a lower response rate than wild-type patients. This is in line with a recently conducted study that found the same relation but only based on the first radiologic assessment [21].

The present results indicate an influence of a high baseline ctDNA level on treatment effect. With each increase in baseline ctDNA level of 1%, the risk of treatment failure increased by 2%. This suggests patients with a high ctDNA level to have a poor prognosis, but a more detailed quantification of the threshold for effect is needed.

LctDNA correlated with a low risk of progression under treatment (Fig. 4). Our findings confirm the results in a recent paper by Garlan et al. [24]. They reported a low ctDNA level at baseline, or a level decreasing below 0.1 ng/ml before cycle two, to indicate long PFS and OS. Tie et al. reported that a reduction in ctDNA level before cycle two correlated with response at the first radiological evaluation and a trend for increased PFS [21]. Our results showed agreement between reduction of ctDNA level and improved PFS, but we did not find an association with response. The difference may be explained by different response rates. It was 52% in the study by Tie et al. and only 31% in the present one. The lower rate may be due to RAS/RAF mutations in all our patients.

The present results are the first to show the relationship between increasing level of ctDNA and progression over the whole treatment course. HctDNA at any time implied a high risk of progression (RR = 4.58). The importance of just one such increase was accentuated by the fact that two consecutive HctDNA measurements had a similar impact on the risk of progression (RR = 4.03). Hence, there does not seem to be evidence as to postpone the change in treatment strategy until a second increase is observed. On the other hand, a stable ctDNA level encourages treatment continuation. The increase appears with a lead time of approximately 2 months, which is relevant in the clinical setting.

The present study has several limitations. First of all, it only comprised patients with RAS/RAF mutated tumors, which, however, applied to around 60% of the patients. Additionally, the sample size of patients ending treatment due to PD was small. The reasons for treatment discontinuation, listed in the flow chart, are believed to be in agreement with clinical practice. The correlations of LctDNA with PFS and HctDNA with progression suggest a predictive value of changes in ctDNA level, but a randomized trial is needed for final proof.

Previous studies conclude that ctDNA is a more reliable marker than carcinoembryonic antigen (CEA) which may even be misleading [21, 26, 27]. Further, CEA is not part of the ESMO guidelines [17] and consequently not included in the present study. We only analyzed the plasma samples from tumor wild-type patients at the time of progression, but it is not likely that patients without mutated DNA at progression would have presented with mutated DNA before start of treatment.

## Conclusions

The present study adds preliminary evidence as to the clinical relevance of ctDNA during treatment. An early decrease holds promise as to a long progression free interval. On the other hand a significant increase gives notice of progression. Thus, ctDNA monitoring may be a step towards precision medicine.

## Additional files

Additional file 1: **Table S1.** Mutations tested in three consecutive rounds of tumor analyses. (JPEG 44 kb)

Additional file 2: Suplementary material. (DOC 35 kb)

Additional file 3: **Table S2.** BioRad PrimePCR ddPCR assays for specific mutations. (JPEG 118 kb)

Additional file 4: **Table S3.** Distribution of mutations in plasma DNA found at baseline. (JPEG 29 kb)

## Abbreviations

CEA: Carcinoembryonic antigen
CI: Confidence interval
CRC: Colorectal cancer
ctDNA: Circulating tumor DNA
ddPCR: Droplet digital polymerase chain reaction
HctDNA: a substantial increase in the ctDNA
HR: Hazard ratio
LctDNA: Low ctDNA
mCRC: Metastatic CRC
NGS: New Generation Sequencing
OS: Overall survival
PD: Progressive disease
PFS: Progression free survival
RR: Relative risk
