# Supplementary figures and images for: Monitoring the effect of first line treatment in RAS/RAF mutated metastatic colorectal cancer by serial analysis of tumor specific DNA in plasma
Source: J Exp Clin Cancer Res. 2018 Mar 12;37:55. doi: 10.1186/s13046-018-0723-5 (PMC5848434; doi:10.1186/s13046-018-0723-5)

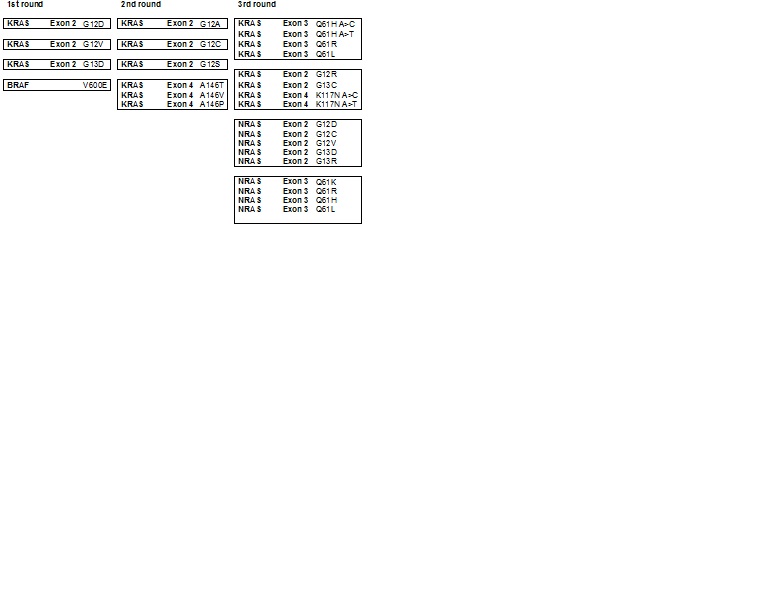

Supplement: Supplementary file 1 — Table S1. Mutations tested in three consecutive rounds of tumor analyses. (JPEG 44 kb) [file 13046_2018_723_MOESM1_ESM.jpg]

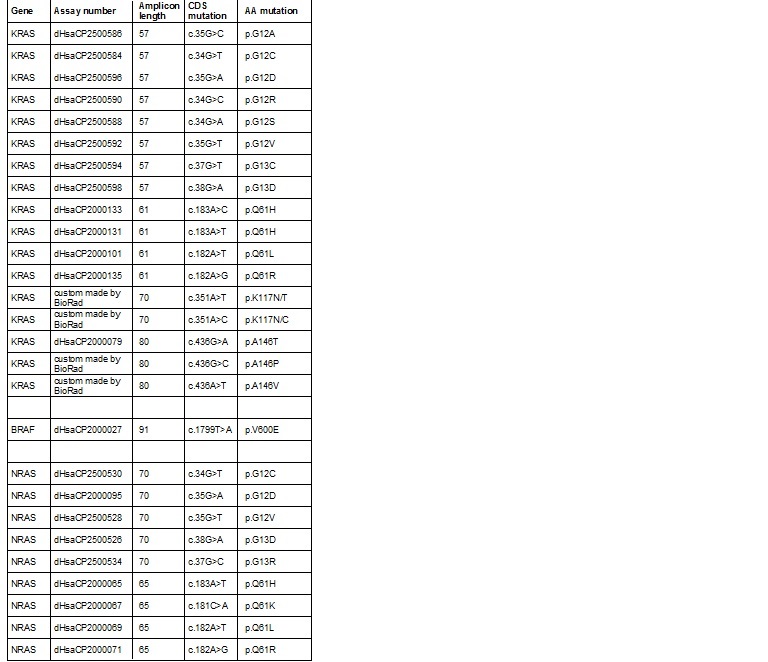

Supplement: Supplementary file 3 — Table S2. BioRad PrimePCR ddPCR assays for specific mutations. (JPEG 118 kb) [file 13046_2018_723_MOESM3_ESM.jpg]

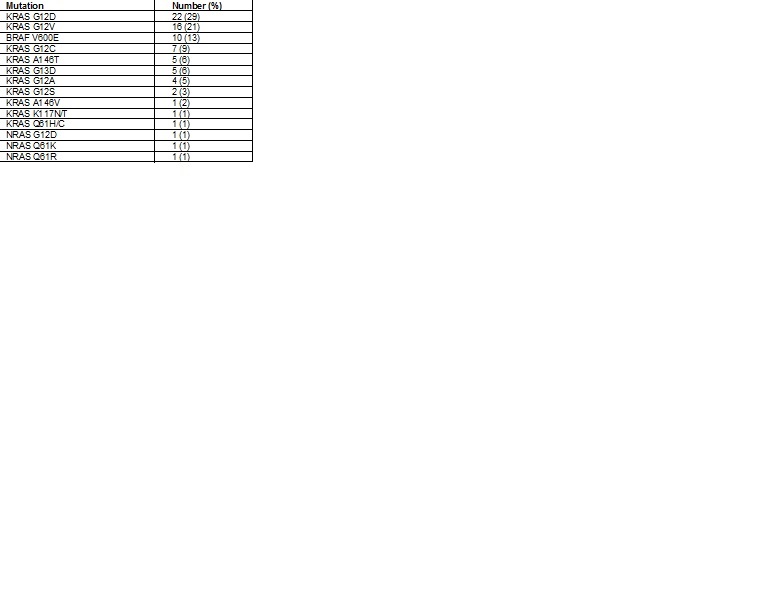

Supplement: Supplementary file 4 — Table S3. Distribution of mutations in plasma DNA found at baseline. (JPEG 29 kb) [file 13046_2018_723_MOESM4_ESM.jpg]
