# Supplementary material for: Monitoring the effect of first line treatment in RAS/RAF mutated metastatic colorectal cancer by serial analysis of tumor specific DNA in plasma
Source: J Exp Clin Cancer Res. 2018 Mar 12;37:55. doi: 10.1186/s13046-018-0723-5 (PMC5848434; doi:10.1186/s13046-018-0723-5)
Supplement: Supplementary file 2 — Suplementary material. (DOC 35 kb) [file 13046_2018_723_MOESM2_ESM.doc]

**Supplementary material**

Inclusion criteria

The inclusion criteria were adenocarcinoma in the colon or rectum, recurrent or primary metastatic disease, measureable disease according to RECIST, planned treatment with chemotherapy (capecitabine and oxaliplatin) and bevacizumab, age above 18 years, and ECOG performance status 0-2. Treatment was discontinued in case of progressive disease (PD), unacceptable toxicity, death, patient request, or as decided by the treating physician.

Tumor analysis

Tumor tissue from the primary site (*N*=134) or metastasis (*N*=4) was formalin fixed and paraffin embedded (FFPE) by routine methods and stored under standard and consistent conditions. Tumor slides were reviewed for selection of paraffin blocks with abundant tumor cells. In some cases laser microdissection was carried out to increase the percentage of cancer cells. Three 15 µm slices of FFPE tumor tissue were subjected to 180 µl incubation buffer and 20 µl protein kinase K overnight at 70°C. Four hundred µl lysis buffer was added to the DNA samples that were purified on the MAXWELL^TM^ 16 LEV instrument using FFPE Plus LEV DNA Purification kit (AS135 Promega, Madison, WI, USA) according to the manufacturer's recommendations. DNA was eluted in 50 µl nuclease free water and further diluted with 500 µl nuclease free water (300 µl if the cancer tissue was from a biopsy due to less material). BioRad ddPCR supermix, PrimePCR ddPCR assays for specific mutations (Bio-Rad®, Table S2) and purified DNA were mixed with oil, and droplets were generated in the Automated Droplet Generator (Bio-Rad®). For multiplex reactions, wild-type assays and assays for each mutation were mixed in equal amounts and 2 µl was used for each 20 µl reaction. Forty cycles of PCR amplification were carried out (initial denaturation at 95°C for 10 min, 40 cycles of 94°C for 30 sec and 55°C for 60 sec, and final extension at 98°C for 10 min), and the samples were analyzed for droplets containing mutated and wild-type DNA in the Droplet Reader QX100 (Bio-Rad®). Quantasoft ddPCR software ver. 1.7 was used for analyzing data. Data was visualized and the concentrations of droplets with a mutation were quantified.

Blood sample analysis

Two nine ml blood samples were collected in EDTA tubes and plasma was isolated by centrifugation at 2,000g for 10 minutes within four hours and stored at - 80°C until use. The plasma was centrifuged again at 10,000g for 10 minutes before purification and Cysteine-rich polycomb-like protein1 (CPP1) DNA fragments were added as exogenous internal control [24]. The DNA was purified from 4 x 1.0 ml plasma on the QIAsymphony SP instrument using the QIAsymphony DSP Virus/Pathogen midi kit (Qiagen, Hilden, Germany) according to the manufacturer’s instructions. Samples with inadequate volume (18/571) were added water. The DNA was eluted in 4 x 110 µl of the supplied buffer and pooled, and qPCR was performed on 20 µl eluate to quantify CPP1 and total cell free DNA (gB2M) as previously described [24]. The remaining eluate was concentrated on an Amicon Ultra 0.5 centrifugal filter unit (Millipore, Billerica, MA, USA) to 20µl and ddPCR was completed in two wells and analyzed for the specific mutation.

Droplet digital PCR

Positive controls for each mutation (gBlocks, IDT, Carolville, IA, USA) or mutated fragments generated according to Spindler et al. [25], genomic donor DNA, and water were included in the analyses as controls. Limit of blank was determined by donor controls and less than two FAM positive droplets were observed in all analyses of the genomic donor DNA controls and plasma DNA from healthy individuals. Therefore, plasma samples with more than two positive FAM droplets in two wells were classified as positive. All analyses were carried out by staff blinded to the clinical status of the patients.

Statistical analysis

Descriptive statistics included median and range of continuous variables, and number and percentage of categorical ones.

Overall survival (OS) was calculated from the date of initiating therapy to the date of death of any cause using the Kaplan-Meier method. Similarly, progression free survival (PFS) was calculated to the date of first reported PD or death of any cause. Patients were censored for PFS at the date of an intervention (e.g. radiofrequency ablation), date of most recent disease evaluation or hospital contact in case of non-PD, or when allocated to a follow-up program without treatment. For the entire study population OS was only censored at the last known date alive. Hazard ratios were estimated by Cox regression.

The independent prognostic impact of mutational status in plasma at baseline was examined by logistic regression with ctDNA included as a continuous variable. Logistic regression analysis was also applied to elucidate the quantitative relationship between HctDNA and progression on treatment. The risk ratio was estimated using a generalized linear model with binary distribution and log link function.

All reported p-values were two-sided and p-values < 0.05 were considered statistically significant.

Statistical analyses were carried out using STATA 14.0 Stata Corp. College Station, TX, USA.
